# Factors associated with unmet dental care needs in Canadian immigrants: an analysis of the longitudinal survey of immigrants to Canada

**DOI:** 10.1186/1472-6831-14-145

**Published:** 2014-12-03

**Authors:** Paola Calvasina, Carles Muntaner, Carlos Quiñonez

**Affiliations:** Faculty of Dentistry & Global Health Division, Dalla Lana School of Public Health, University of Toronto, Toronto, ON Canada; Bloomberg Faculty of Nursing, Institute for Global Health Equity and Innovation, Dalla Lana School of Public Health, University of Toronto, Toronto, ON Canada; Discipline of Dental Public Health, Faculty of Dentistry, University of Toronto, Toronto, ON Canada

**Keywords:** Dental care, Immigrants, Longitudinal studies, Canada

## Abstract

**Background:**

Immigrants are often considered to have poorer oral health than native born-populations. One possible explanation for immigrants’ poor oral health is lack of access to dental care. There is very little information on Canadian immigrants’ access to dental care, and unmet dental care needs. This study examines predictors of unmet dental care needs among a sample of adult immigrants to Canada over a three-point-five-year post-migration period.

**Methods:**

A secondary data analysis was conducted on the Longitudinal Survey of Immigrants to Canada (LSIC). Sampling and bootstrap weights were applied to make the data nationally representative. Simple descriptive analyses were conducted to describe the demographic characteristics of the sample. Bivariate and multiple logistic regression analyses were applied to identify factors associated with immigrants’ unmet dental care needs over a three-point-five-year period.

**Results:**

Approximately 32% of immigrants reported unmet dental care needs. Immigrants lacking dental insurance (OR = 2.63; 95% CI: 2.05-3.37), and those with an average household income of $20,000 to $40,000 per year (OR = 1.62; 95% CI: 1.01-2.61), and lower than $20,000 (OR = 2.25; 95% CI: 1.31-3.86), were more likely to report unmet dental care needs than those earning more than $60,000 per year. In addition, South Asian (OR = 1.85; CI: 1.25-2.73) and Chinese (OR = 2.17; CI: 1.47-3.21) immigrants had significantly higher odds of reporting unmet dental care needs than Europeans.

**Conclusions:**

Lack of dental insurance, low income and ethnicity predicted unmet dental care needs over a three-point-five-year period in a sample of immigrants to Canada.

## Background

Over the last several decades, Canada has become an increasingly multicultural society. Approximately 250,000 immigrants enter Canada each year, arriving from Asia and Pacific Rim countries (49%), Africa and the Middle East (24%), the United Kingdom and Europe (13%), South and Central America (11%), and the United States (3%) [1]. Yet unfortunately, there is very limited information on the oral health of adult immigrants in Canada. Previous cross-sectional studies have suggested that, in general, immigrants have poorer oral health status than the Canadian population [2–5]. Among the possible explanations for immigrants’ poor oral health is lack of access to dental care.

Access to care is a multidimensional concept that has often been defined as the ability to obtain needed health care [6]. The inability to obtain needed care is referred to as an ‘unmet health care need’, a concept commonly used in health service research to indicate barriers to care [7]. In dentistry, unmet dental care needs have been correlated with poor oral health and poor dental service utilization [8, 9]. In many studies in the US, unmet dental care needs have also been used to measure difficulties with access to dental care due to service costs, or lack of insurance in many studies in the US [10–13]. Therefore, in this study, unmet dental care needs was used as an indicator of barriers to gaining access to dental care.

In turn, very little is known about immigrants’ access to dental care in Canada. Earlier studies have provided equivocal findings. For instance, while Bedos et al. [14] reported lower rates of dental service utilization among immigrants in comparison with Canadian-born populations, Newbold and Patel [15] demonstrated that immigrants have a higher rate of dental utilization than Canadian-born populations. However, the latter also reported that, compared with native-born Canadians, immigrants were more likely to consult a dentist for treatment rather than for preventive reasons [15]. Recently, an analysis of the Canadian Health Measure Survey (CHMS 2007-2009) revealed that immigrants have a higher risk of reporting various negative outcomes associated with poor oral health and having access to dental care [8, 16, 17] than Canadians. For instance, immigrants had a higher prevalence of self-reported untreated dental conditions [16], a lower prevalence of dental insurance coverage [16], and were more likely to have untreated periodontal disease [17]. In addition, although cost barriers such as low income and a lack of dental insurance are the two dominant predictors of limited access to dental care among the overall Canadian population [8, 18–20], immigrants have higher odds of reporting cost barriers to dental care than those born in Canada [8].

Other factors associated with immigrants’ access to dental care include language and cultural barriers [21, 22] or unfamiliarity with the health care system. Furthermore, access to dental care can vary across different ethnic groups [23]. A recent report on access to health care in Ontario revealed that over half of non-European immigrants had not visited a dentist in the previous 12 months compared to less than 35% of Europeans [23].

Understanding the unmet dental care needs of immigrants to Canada is important, in order to plan effective policy interventions with the aim of eliminating potential barriers to care, thus offering opportunities for immigrants to fully contribute to the Canadian economy. This study examines predictors of unmet dental care needs among a sample of recent immigrants to Canada over a three-point-five-year period.

## Methods

### Data source

**Figure 1:**
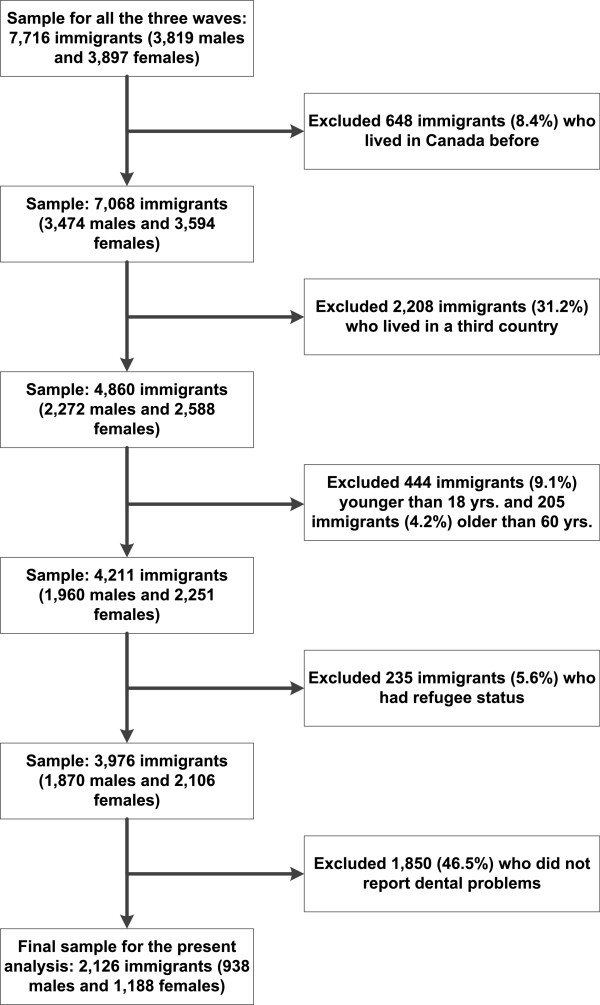
**Sample size for this analysis of the longitudinal survey of immigrants to Canada (2001-2005).**

This study uses three waves of Statistics Canada’s Longitudinal Survey of Immigrants to Canada (LSIC) to study immigrants’ unmet dental care needs. The survey collected information in more than 15 languages on socio-economic status, housing, language skills, values and social attitudes, social support, health status, access and utilization. Measures of access to, and utilization of dental services were included in the LSIC Health Module. Data were collected at six months (wave 1), two years (wave 2) and four years (wave 3) after immigration. The LSIC target population was derived from a sample of immigrants: 1) who arrived in Canada between October 2000 and September 2001; 2) were aged 15 years or older at time of landing; and 3) landed from abroad, and applied through a Canadian Mission abroad. A two-stage stratified sampling method was used to select the survey respondents. The first stage involved the selection of an immigrant unit (individual, couples or families) from the administrative database of Citizenship and Immigration Canada. The second stage involved the selection of one member from the immigrant unit, aged 15 years or older at the time of landing. Interviews were conducted in person and by telephone. A total of 7,716 respondents completed the three waves of interviews, representing a cohort of 157,600 immigrants [24]. Our study sample included non-refugee immigrants aged 18-60 years old, who left their country of origin directly to immigrate to Canada (i.e., they did not live in Canada or in a third country before participating in the LSIC study) and who reported an unmet dental care need over the years after immigrating to Canada. Thus, our final study sample comprised 2,126 immigrants. Figure 1 shows details of the sample selection criteria. We excluded immigrants who had lived in Canada before immigrating, as well as those who had lived in a third country before participating in the LSIC, because we were interested in examining the effect of the first process of immigration on immigrants’ ability to have access to dental care in Canada. Previous exposure to acculturation would possibly impact new Canadian immigrants’ perception of dental problems and unmet dental care needs. Data were accessed from Statistics Canada Research Data Centre (RDC) at the University of Toronto. Permission to access the data was granted by a Statistics Canada Subject Matter Expert based on the relevance of the research methods, and the expertise of the research team to carry out the proposed research. There was no need to obtain ethical approval for this study. According to Canadian National Ethics regulations [25] data held by Statistics Canada is legally accessible and appropriately protected by law.

### Study variables

Self-reported unmet dental care needs were used as a proxy for barriers to having access to dental care. This study outcome was obtained using the following survey questions: “Since your last interview, have you had any dental problems for which you did not receive dental care?” (yes /no). This question was asked at wave 2 and wave 3. Due to the small proportion of variability in self-reported unmet dental care needs between the two waves, we developed a summary outcome variable by adding the responses across the waves. Thus, the outcome was categorized as “yes” if the immigrants reported having any unmet dental care needs over a three-point-five-year period, otherwise it was categorized as “no”.

Covariates examined in bivariate and logistic regression analyses for their relationship with unmet dental care needs over a three-point-five-year period were selected based on: 1) information collected and available in the LSIC; and 2) variables that have previously been explored in the dental literature on immigrants’ access to dental care [2–6, 9–14]. Thus, three categories of independent variables were considered: 1) socio-demographic; 2) socio-economic; and 3) official language proficiency.

### Socio-demographic variables

Socio-demographic variables included age, sex, marital status and ethnicity. Age was classified in the following groups: 20-29, 30-39, 40-49, and ≥ 50. Marital status was dichotomized as married or not married (single, divorced, widowed). Ethnicity was obtained on the basis of ethnic origin, as defined by Statistics Canada [26]. We selected this variable to capture ethnic disparities in immigrants’ access to dental care in Canada. This variable was categorized as: 1 = Arabic, African and Middle Eastern (Arabic world, African continent and Middle East Asia); 2 = South Asian (India, Pakistan, and Sri Lanka); 3 = Chinese (Mainland China, Taiwan, Hong Kong); 4 = East Asia (Korean, Japanese and other East Asian countries); 5 = Latin American and Caribbean (Caribbean, Mexico, Central and South America).

### Socio-economic variables

Socio-economic variables included education, history of social assistance, dental insurance coverage, employment status and average household income. Education was categorized as having a college, university degree and more vs. a high school diploma or less. History of social assistance was categorized as yes/no. Dental insurance coverage was asked only at year four (wave 3), and categorized as yes/no.

Employment status and average household income were the only two variables that varied across time. Thus, we created summary variables that accounted for time variability and reflected the cross-sectional nature of the analysis. Employment status was categorized as always being employed vs. always/sometimes unemployed over time. The summary of the household income variable was derived in the following way. First, we created a variable containing the average income across the three waves, and then we calculated the mean and standard deviation of this variable. Since the standard deviation rounded to $20,000 a year, and $40,000 a year is a good proxy for the threshold income of the working poor in Canada [27], we created four categories for the income variable that included: ≥$60,000; $40,000-$60,000; $20,000-40,000; ≤$20,000.

### Official language proficiency variables

Official language proficiency was assessed through self-reported proficiency in writing, reading, and speaking in English or French (cannot write/speak/read, poorly, fairly well, well, very well). We recoded responses to these questions as poor, moderate and good/very good.

### Statistical analysis

Simple descriptive analyses were conducted to describe the demographic characteristics of the sample of immigrants. Bivariate analyses were performed to examine the relationship between each socio-demographic, socio-economic and official language variables and unmet dental care needs. Statistical significance was set at the 0.05 level. Before estimating the multiple logistic regression models, we examined collinearity amongst and between each of the variables, using the variance inflation factor (VIF). Only those variables with a VIF equal to or less than three were entered into the model. Multiple logistic regression analyses were conducted to assess the independent association between unmet dental care needs and the socio-demographic, socio-economic and official language variables. Two logistic regression models were then estimated. In model 1, we entered education, income, history of social assistance, and employment status effects on unmet dental care needs controlling for age, sex, ethnicity, marital status and official language proficiency. In Model 2, we entered the dental insurance variable into Model 1, in order to explore the effect of lack of dental insurance on the odds ratio of the other variables and the outcome variable. We used Akaike Information Criteria (AIC) to compare the fit of models while accounting for additional variables in the models [28]. Sample weights and 1,000 bootstrap weights, as specified by Statistics Canada were applied in all statistical analysis. We performed all analysis using the software program STATA version 12 (Stata Corp. College Station, Texas, US).

## Results

The final sample included 2,126 participants, representing almost 47, 050 immigrants to Canada when weighted, out of 157,600 immigrants who resided in Canada from 2001 to 2005 [29]. Table 1 shows that the sample consisted of a majority of women (55.9%), married (87.5%), aged between 30-39 years (47.1%). South Asian and Chinese immigrants represented more than 50% of the total sample, whereas immigrants from Latin American and Caribbean countries corresponded to 5% of the total sample. Although the majority of immigrants were highly educated, with college or university degrees and beyond, more than 64% reported an average household income of less than $40,000 a year. Approximately 43% of the sample rated their official language proficiency (English/French) as fair, and around 40% rated their language proficiency as poor. Almost two-thirds of immigrants reported having dental insurance at year four. Approximately 32.3% of immigrants reported unmet dental care needs over time in a three-point-five-year period.

**Table 1:** **Demographic characteristics of the study sample (weighted proportions)**

	(%)
Sex	
Male	44.1
Female	55.9
Age	
20-29	28.9
30-39	47.1
40-49	18.2
≥ 50	5.8
Ethnicity	
European	20.6
Arabic/African/Middle Eastern	14.2
South Asian	22.6
Chinese	25.1
East Asian	12.5
Latin American/Caribbean	5.0
Marital Status	
Married	87.5
Not married	12.5
Highest level of education	
College, university degree and more	64.4
High school and less	35.6
Average household income	
≥ $60,000	10.2
$40,000- $60,000	24.0
$20,000-$40,000	45.1
≤ $20,000	20.7
History of social assistance	
No	87.7
Yes	12.3
Official language proficiency (English/French)	
Poor	41.4
Fair	43.1
Good/very good	15.5
Employment status	
Always employed	35.9
Always/sometimes unemployed	64.1
Unmet dental care needs	
Yes	32.3
No	67.7
Dental insurance	
Yes	61.1
No	38.9

In the bivariate analysis, immigrants with less than high school education (OR = 1.72; 95% CI: 1.35-2.20), an average household income of less than $20,000 (OR = 3.96; 95 CI: 2.56-6.11), with a history of social assistance (OR = 1.47; 95% CI: 1.09-1.98), experience of unemployment (OR = 1.78; 95% CI: 1.44-2.21), and lacking dental insurance (OR = 3.21; 95% CI: 2.63-3.92), were more likely to have had an unmet dental care need. Very good/good official language proficiency was inversely correlated with unmet dental care needs over time (OR = 0.61; 95% CI: 0.45-0.81). Other correlates of unmet dental care needs included age and ethnicity (Table 2). Sex and marital status were not associated with unmet dental care needs over a three-point-five-year period.

**Table 2:** **Unadjusted associations with unmet dental care needs among immigrants sampled in this study (weighted proportions)**

	Unmet dental care needs		OR	95% CI	p-value
	Yes (n = 686, 32.3%)	No (n = 1440, 67.7%)			
Sex					
Male	293 (31.3%)	644 (68.7%)	Ref		
Female	393 (33.0%)	796 (67.0%)	1.08	0.89-1.32	
Age					
20-29	169 (27.5%)	446 (72.5%)	Ref		
30-39	323 (32.3%)	679 (67.7%)	1.25	0.99-1.58	
40-49	138 (35.8%)	248 (64.2%)	**1.47**	**1.11-1.94**	**< 0.001**
≥ 50	55 (44.7%)	68 (55.3%)	**2.12**	**1.43-3.17**	**< 0.001**
Ethnicity					
European	84 (19.7%)	342 (80.3%)	Ref		
African/Arabic/Middle Eastern	104 (35.4%)	190 (64.6%)	**2.23**	**1.58-3.16**	**< 0.001**
South Asian	175 (37.3%)	294 (62.6%)	**2.43**	**1.81-3.26**	**< 0.001**
Chinese	229 (44.0%)	292 (56.0%)	**3.20**	**2.36-4.33**	**< 0.001**
East Asian	53 (20.4%)	207 (79.5%)	1.05	0.70-1.57	
Latin American	24 (23.5%)	80 (76.5%)	1.25	0.74-2.12	
Marital status					
Married	607 (32.6%)	1253 (67.3%)	Ref		
Not married	79 (29.6%)	184 (70.4%)	0.87	0.64-1.18	
Highest level of education outside Canada					
College/University degree and more	547 (30.3%)	1256 (69.7%)	Ref		
High school or less	138 (42.8%)	184 (57.2%)	**1.72**	**1.35-2.20**	**< 0.001**
Average household income					
≥ $ 60,000	38 (18.3%)	169 (81.7%)	Ref		
$40,000- $60,000	101 (20.7%)	388 (79.3%)	1.17	0.75-1.83	
$20,000-$40,000	327 (35.7%)	589 (64.3%)	**2.49**	**1.65-3.74**	**< 0.001**
≤ $20,000	197 (46.9%)	223 (53.1%)	**3.96**	**2.56-6.11**	**< 0.001**
History of social assistance					
No	506 (31.2%)	1115 (68.8%)	Ref		
Yes	91 (40.0%)	136 (60.0%)	**1.47**	**1.09-1.98**	**0.011**
Official language proficiency (English/French)					
Poor	316 (39.4%)	487 (60.6%)	Ref		
Fair	244 (29.1%)	593 (70.8%)	**0.63**	**0.51-0.78**	**< 0.001**
Good/very good	85 (28.3%)	216 (71.7%)	**0.61**	**0.45-0.81**	**< 0.001**
Employment status					
Always Employed	187 (24.5%)	577 (75.53%)	Ref		
Unemployed once or more	449 (36.6%)	863 (63.4%)	**1.78**	**1.44-2.21**	**< 0.001**
Dental insurance					
Yes	289 (22.29%)	1007 (77.71%)	Ref		
No	396 (47.96%)	430 (52.04%)	**3.21**	**2.63-3.92**	**< 0.001**

In Model 1 of the multiple logistic regression analysis, average household income, educational level, ethnicity and age remained statistically significant after controlling for all covariates (Table 3). In Model 2, with the inclusion of the dental insurance variable, the odds ratio of age became not significant and in general, there was a reduction in the odds ratio of the other significant variables from Model 1. Model 2 showed that a lack of dental insurance (OR = 2.63; 95% CI 2.05-3.37) was strongly correlated with unmet dental care needs (Model 2). Furthermore, immigrants with an average household income between $20,000 to $40,000 (OR = 1.62; 95% CI: 1.01-2.61), and lower than $20,000 (OR = 2.25; 95% CI: 1.31-3.86), were more likely to report unmet dental care needs than those earning over $60,000. Moreover, immigrants from South Asian (OR = 1.85; 95% CI: 1.25-2.73) and Chinese (OR = 2.17; 95% CI: 1.47-3.21) ethnic origins were more likely to report unmet dental care needs than Europeans over the three-point-five-year period.

**Table 3:** **Associations of individual socio-demographic and economic factors and unmet dental care needs obtained from a sample of immigrants: Longitudinal Survey of Immigrants to Canada (2001-2005)**

	Model 1, OR (95% CI)	Model 2, OR (95% CI)	p-value
Sex			
Male (Ref)	1.00	1.00	
Female	1.05 (0.82-1.35)	1.08 (0.83-1.40)	
Age			
20-29(Ref)	1.00	1.00	
30-39	1.17 (0.88-1.57)	1.22 (0.91-1.64)	
40-49	**1.53 (1.07-2.18)**	1.39 (0.96-2.01)	
≥ 50	1.62 (0.93-2.81)	1.12 (0.63-2.00)	
Ethnicity			
European origins (Ref)	1.00	1.00	
Arabic/African/West Asian	1.29 (0.83-1.99)	1.21 (0.77-1.91)	
South Asian	**1.93 (1.31-2.83)**	**1.85 (1.25-2.73)**	**< 0.001**
Chinese	**2.12 (1.45-3.12)**	**2.17 (1.47-3.21)**	**< 0.001**
East Asian	1.10 (0.67-1.80)	1.11 (0.67-1.83)	
Latin American/Caribbean	0.95 (0.49-1.83)	0.91 (0.46-1.77)	
Marital status			
Married (Ref)	1.00	1.00	
Not-married	1.01 (0.68-1.51)	1.04 (0.69-1.57)	
Highest level of education			
College, University and More (Ref)	1.00	1.00	
High school and less	**1.64 (1.15-2.32)**	**1.53 (1.07-2.20)**	**0.020**
Average household income			
≥ $ 60,000	1.00	1.00	
$40,000 - $60,000	0.89 (0.54-1.48)	0.90 (0.54-1.50)	
$20,000 - $40,000	**1.81 (1.13-2.89)**	**1.62 (1.01-2.61)**	**0.046**
≤ $ 20,000	**2.83 (1.67-4.79)**	**2.25 (1.31-3.86)**	**< 0.001**
History of social assistance			
No (Ref)	1.00	1.00	
Yes	1.17 (0.76-1.81)	1.11 (0.71-1.74)	
Official language proficiency			
Poor (Ref)	1.00	1.00	
Moderate	0.83 (0.63-1.09)	0.96 (0.72-1.27)	
Good/very good	0.90 (0.58-1.39)	0.98 (0.63-1.53)	
Employment status			
Always employed	1.00	1.00	
Always/sometimes unemployed	1.30 (0.98-1.71)	1.19 (0.90-1.58)	
Dental insurance			
Yes		1.00	
No		**2.63 (2.05-3.37)**	**< 0.001**
AIC		**1,86897**	

## Discussion

This study is the first to explore the issue of unmet dental care needs, using a representative sample of immigrants to Canada, who had not lived in a third country before participating in the LSIC. Our findings suggested that approximately 32.3%, roughly fifteen thousand immigrants reported an unmet dental care needs over a three-point-five-year period. This finding reflects those of Thompson [8], who found that 31.2% of immigrants who had been living in Canada for less than ten years reported avoided visiting a dental professional due to cost. This was a significantly higher proportion than the proportion of immigrants who had been living in Canada for longer than ten years (17.5%).

After controlling for all the independent variables, a lack of dental insurance and an average household income lower than $40,000 a year were the main predictors of unmet dental care needs. Importantly, a lack of dental insurance was the strongest predictor of immigrants’ unmet dental care needs (i.e., access to dental care). Those who lacked dental insurance, on average, had higher odds of reporting unmet dental care needs than immigrants with a household income between $20,000-$40,000 and lower than $20,000.

In addition, the provision of dental insurance for immigrants with an average household income between $20,000-$40,000 and lower than $20,000 significantly reduced the odds of reporting unmet dental care needs. For instance, for immigrants with dental insurance the odds of reporting unmet dental care needs was 0.38 (OR = 1/2.63 = 0.38), while the odds of immigrants with a household income between $20,000-$40,000 was 1.65, and for an income lower than $20,000 it was 2.25. Consequently, providing dental insurance for immigrants in the former and latter income groups reduced their odds of reporting unmet dental care needs to 0.62 (OR = 1.65*0.38 = 0.62) and 0.86 (OR = 2.25*0.38 = 0.86), respectively. Thus, dental insurance may contribute significantly to eliminating income disparities in immigrants’ unmet dental care needs in the predominantly private fee-for-service Canadian dental care system. This finding corroborates those of Ramraj [17] and Thompson [7], whose analyses of the CHMS, which included immigrants, found that dental insurance was a more important determinant of having unmet dental care needs than income. Our finding is also consistent with that of Newbold and Patel [15], who identified that having dental insurance was a predictor of immigrants’ dental service utilization. The present study adds further support to the fundamental role of dental insurance in mitigating access to dental care in Canada. Our findings may also be generalized to similar immigrant samples in Australia and New Zealand, two countries with immigration flows [30] comparable with those of Canada.

Ethnicity was significantly associated with unmet dental care needs over a three-point-five-year period of observation for some, but not all groups. When compared with Europeans, Chinese and South Asian immigrants were more likely to report unmet dental care needs. This finding is consistent with previous Canadian literature showing that European respondents report higher dental care utilization than other ethnic groups, including Asian and South Asian immigrants [15, 23]. One possible explanation for Europeans’ having potentially better access to dental care is related to evidence showing that European immigrants have higher earnings than other ethnic immigrant groups in Canada [29, 31], an advantage that probably improves their ability to have access to dental care. Moreover, South Asian and Chinese immigrants are among the most disadvantaged individuals in the Canadian labor market [29, 31]. For instance, analysis of the 2006 Census indicated that Chinese and South Asian immigrants whose employment status was matched to that of White immigrants earned 8% and 30% less, respectively, than their White counterparts. These differences in earnings from employment may reflect forms of racial discrimination in the Canadian Labor market, which forces racialized (i.e., non-European) immigrants into precarious employment, lower earnings and poverty [31], all aspects that potentially explain ethnic disparities in unmet dental care needs found in this study.

### Limitations

This study has several limitations. Firstly, the data did not allow us to perform a longitudinal analysis because: 1) we did not have information on unmet dental care needs at wave 1; and 2) there was no significant change in the proportion of immigrants who reported unmet dental care needs between waves 2 and 3. Thus, we performed a cross-sectional analysis, which prevented causal determination. However, cross-sectional studies offer an optimal exploratory analysis of factors influencing access to dental care in Canada in a population about whom very little is known. Secondly, our results rely on immigrants’ self-reports of unmet dental care needs rather than on direct observation. Self-reports have been found to provide different assessment from those of clinically determined standards [17, 32]. However, it is often an underestimation of clinically determined treatment needs [17]. Thus, it is possible that more than 32.3% of immigrants in the survey had clinically relevant unmet dental care needs. Thirdly, our results are based on a sample of immigrants who immigrated in 2001. While the immigrant population in Canada has grown tremendously since that time, this study still reflects the most recent and accurate estimate of unmet dental care needs among a representative sample of the immigrant population in Canada.

### Policy implications

Our findings suggest that financial barriers, in particular dental insurance, represent the predominant factors in explaining immigrants’ overall unmet dental care needs over time. Although financial barriers are also significant determinants of access to dental care among the general Canadian population [8, 18–20], it is clear that immigrants experience greater cost-prohibitive barriers to having access to dental care than non-immigrant Canadians [13]. Thus, our findings underscore the need for improving immigrants’ dental insurance coverage through the expansion of public dental insurance programs that would ensure subsidized/free of charge access to dental care for adult immigrants in Canada. This is the best option for improving oral health equity in Canada. Another alternative would be to develop programs that would direct immigrants to permanent employment that includes dental insurance coverage. These two policy interventions may constitute important components of efforts to improve immigrants’ access to dental care. Specific attention should also be paid to South Asian and Chinese immigrants who were found to be at greater risk of unmet dental care needs.

## Conclusion

Our research found that financial barriers were associated with immigrants’ unmet dental care needs. Immigrants without dental insurance and with low income were more likely to have unmet dental care needs when compared to their counterparts. In addition, Chinese and South Asian immigrants had higher odds of reporting unmet dental care needs than Europeans.

## Abbreviations

LSIC: Longitudinal Survey of Immigrants to Canada
CHMS: Canadian Health Measures Survey
RDC: Research Data Centre
VIF: Variance Inflation Factor
AIC: Akaike Information Criteria.
